# Oral Ranitidine as an Immunomodulatory Agent in the Treatment of Molluscum Contagiosum: A Pediatric Case Report

**DOI:** 10.7759/cureus.98310

**Published:** 2025-12-02

**Authors:** Binod Kumar, Kiran Kumre, Madhavi Thombare, Ratan Kumar, Tulika Anand

**Affiliations:** 1 Dermatology, Tata Main Hospital, Jamshedpur, IND; 2 Pediatrics, Tata Main Hospital, Jamshedpur, IND; 3 Pediatrics, Manipal Tata Medical College, Manipal Academy of Higher Education (MAHE), Jamshedpur, IND; 4 Dermatology, Manipal Tata Medical College, Manipal Academy of Higher Education (MAHE), Jamshedpur, IND

**Keywords:** cryotherapy ablation, molluscum contagiosum, pediatric dermatology, poxviridae, ranitidine

## Abstract

Molluscum contagiosum (MC) is a common viral skin infection seen predominantly in children. While usually self-limiting, concerns over transmissibility and cosmesis often prompt active treatment. Traditional methods such as curettage, cryotherapy, and chemical agents may cause pain or scarring, limiting their use in young children. This case report highlights the successful treatment of MC with oral ranitidine, an H2-receptor antagonist known for its immunomodulatory properties. A six-year-old girl with multiple umbilicated papules on both buttocks achieved complete lesion clearance after eight weeks of oral ranitidine therapy at 5 mg/kg/day. No recurrence was observed over a six-month follow-up. Given its favorable safety profile and immunostimulatory potential, ranitidine may represent a viable, non-invasive therapeutic option for extensive or recurrent MC in immunocompetent pediatric patients.

## Introduction

Molluscum contagiosum (MC) is a benign cutaneous viral infection, primarily affecting young children. It is caused by molluscum contagiosum virus (MCV) which is a double-stranded DNA virus belonging to the *Poxviridae* family. Clinically, MC presents as multiple, firm, dome-shaped papules with a central umbilication, typically pink or skin-colored in appearance. Most lesions resolve spontaneously within 6-12 months in immunocompetent individuals [1]. Dedicator of cytokinesis 8 (DOCK8) deficiency is a genetic disorder affecting the migration of dendritic and specialized T cells in the skin, which can also lead to extensive lesions in MC [2]. Despite this benign course, active treatment is frequently sought due to cosmetic concerns, potential for autoinoculation, and risk of transmission.

Conventional therapies such as cryotherapy, curettage, chemical destruction, and topical keratolytics can be painful and may cause adverse effects such as scarring or hyperpigmentation [3]. Such modalities are less acceptable in pediatric populations where cooperation is limited, prompting the need for safe, non-invasive alternatives. Here, we present a pediatric case of MC treated successfully with oral ranitidine.

## Case presentation

A six-year-old girl presented to the dermatology outpatient department with multiple, non-pruritic, raised lesions over both buttocks for three months. Initially sparse, the lesions gradually increased in number, with approximately 25 lesions on each side at presentation. The child was otherwise healthy and had no history of immunodeficiency, atopy, or similar lesions among family members.

**Figure 1:**
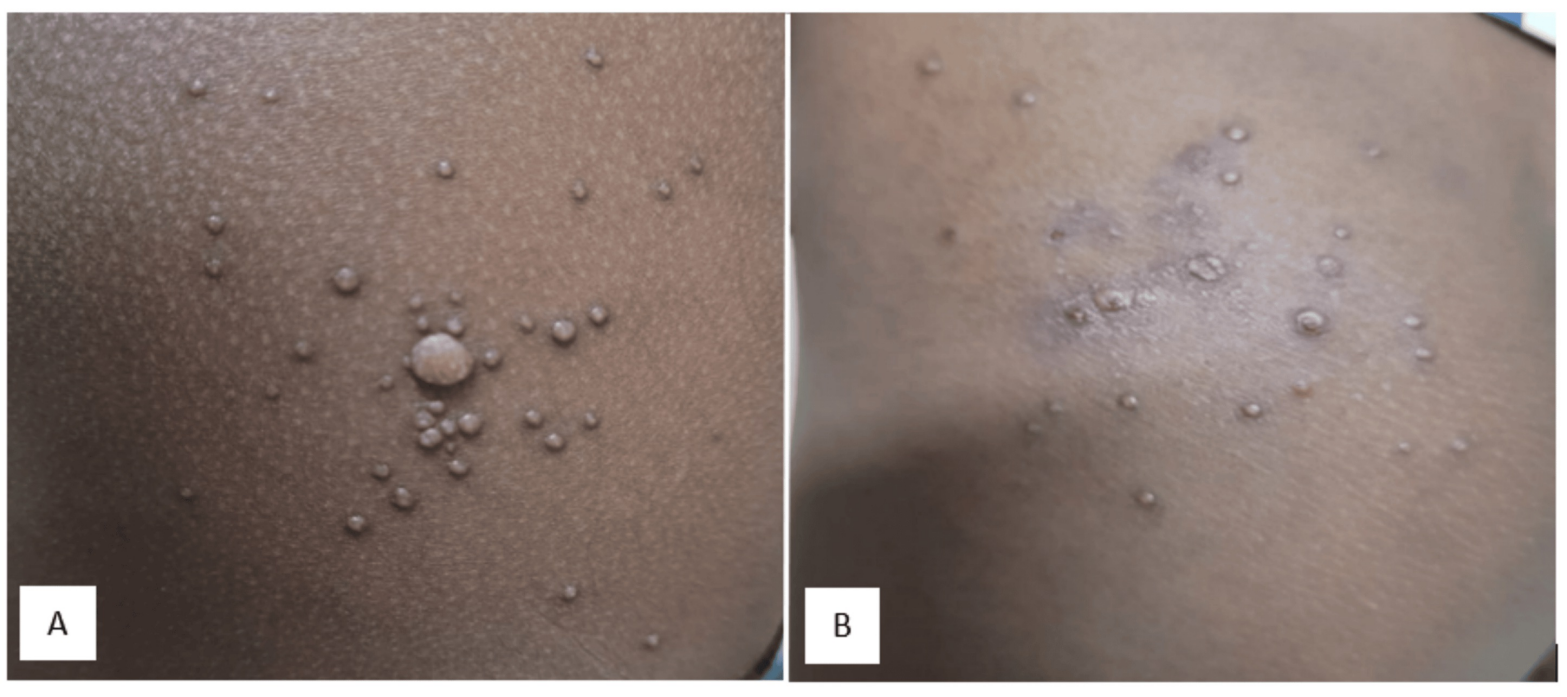
(A) Multiple umbilicated shiny papules over both buttocks at presentation. (B) Lesions fading after two weeks of treatment

Physical examination revealed multiple umbilicated, shiny papules ranging from 1 mm to 5 mm in diameter (Figure 1). Lesions were discrete, flesh-colored, and free of discharge or infection. Systemic examination was normal.

**Figure 2:**
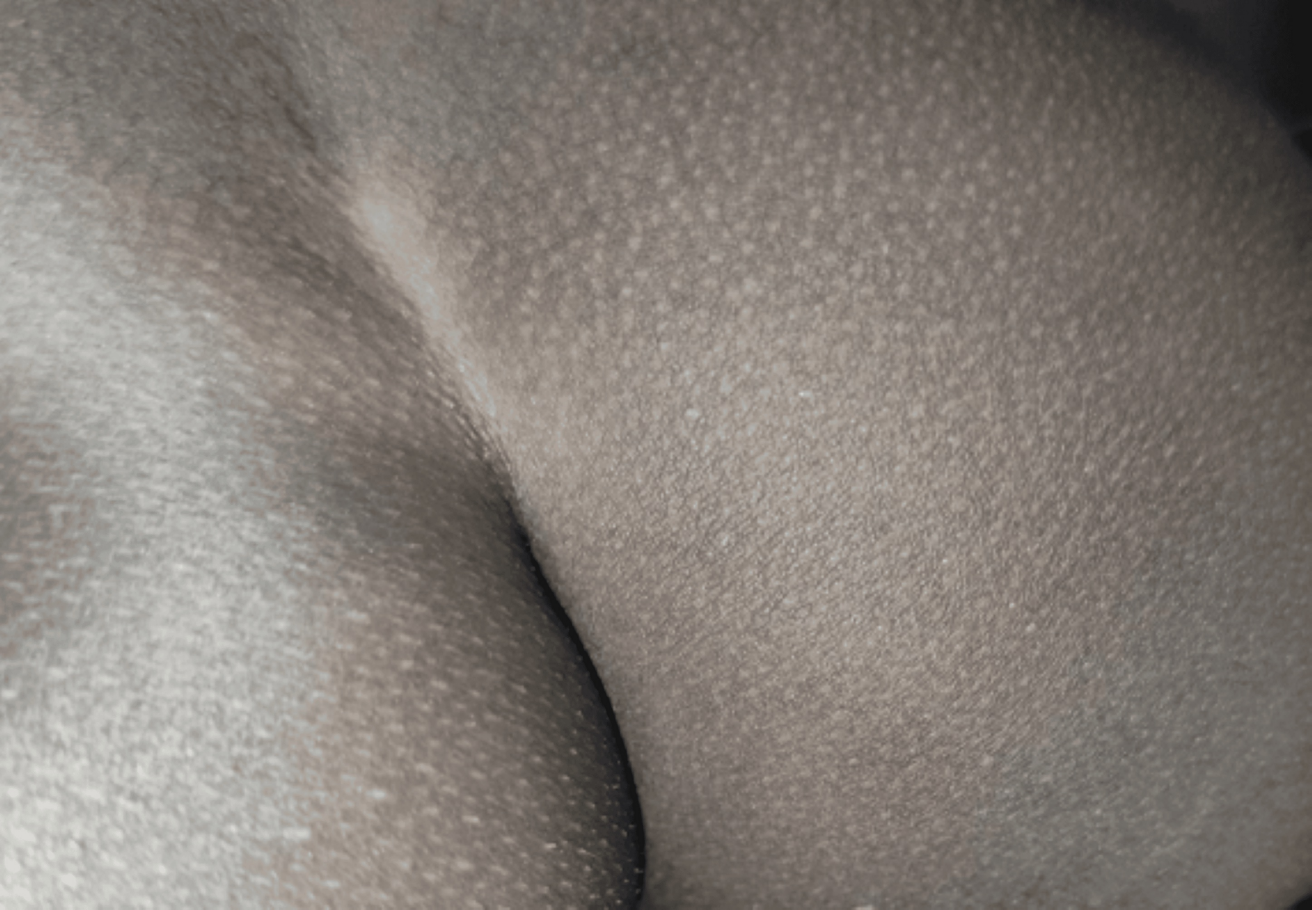
Disappearance of papular lesions after eight weeks of treatment

With a clinical diagnosis of MC, therapeutic choices discussed with parents included radiofrequency or laser excision and oral ranitidine. The child's parents opted for the latter. Oral ranitidine was prescribed at a dose of 5 mg/kg/day, divided into two daily doses for eight weeks. By the end of the treatment period, all lesions had resolved completely (Figure 2).

Follow-ups at two-week intervals and later at three and six months revealed no recurrence or adverse effects. The patient remained otherwise healthy throughout therapy.

## Discussion

Although MC often resolves spontaneously, therapeutic intervention is justified in cases of psychosocial distress, rapidly spreading lesions, or those with secondary infection. None of the existing treatments guarantees complete resolution without risks of discomfort or scarring, especially in pediatric patients [4,5]. Treatment options in children include the use of local anesthetics, such as topical lidocaine; cantharidin; at-home therapies like imiquimod, retinoids, and alpha-hydroxy acids; curettage of visible lesions; and laser and cryosurgery [6]. Cryotherapy, curettage, chemical destruction, and topical keratolytics can be painful, hence poorly tolerated by children, and may cause adverse effects such as scarring or hyperpigmentation. If left untreated, MC can lead to molluscum dermatitis in 10% of patients, which allows further cutaneous spread of the virus [7]. Hence, despite being self-limiting, there is consensus that MC should be actively treated [8].

Ranitidine is a histamine H2-receptor antagonist traditionally used for gastrointestinal conditions. But emerging data indicate that it also exhibits immunomodulatory functions. It can increase CD4+ lymphocytes, decrease CD8+ lymphocytes, and enhance the secretion of interferon-gamma, which augments the function of natural killer cells, thereby boosting the host's antiviral defence. These immune mechanisms may explain its efficacy in MC and related viral disorders [9-11].

In the presented case, ranitidine therapy resulted in full recovery within eight weeks without recurrence for six months post-treatment. This response supports previous evidence that oral H2 blockers may enhance cell-mediated immunity against poxvirus infections. Although spontaneous resolution cannot be entirely excluded, the consistent timing and durability of response suggest a therapeutic contribution from ranitidine. 

Controlled clinical trials comparing ranitidine, cimetidine, and placebo are necessary to confirm these findings. Given the minimal adverse profiles of these agents, H2 blockers may serve as low-risk alternatives when invasive treatments are contraindicated or undesirable.

## Conclusions

Oral ranitidine may represent a simple, safe, and effective therapeutic option for widespread or recurrent MC in immunocompetent children. It offers the advantages of non-invasiveness, minimal side effects, and excellent tolerability. When procedural interventions are impractical or contraindicated, ranitidine can serve as a valuable adjunct or alternative therapy. Continued clinical evaluation of this immunomodulatory approach may help establish its place in the management paradigm for MC.
